# Goat miR-92a-3p Targets *APOL*6 Gene to Regulate the Differentiation of Intramuscular Precursor Adipocytes

**DOI:** 10.3390/genes15010057

**Published:** 2023-12-30

**Authors:** Wuqie Qubi, Jianying Zheng, Youli Wang, Guishan Xu, Yanyan Li, Yan Xiong, Yong Wang, Wei Liu, Yaqiu Lin

**Affiliations:** 1 Key Laboratory of Qinghai-Tibetan Plateau Animal Genetic Resource Reservation and Utilization of Education Ministry, Southwest Minzu University, Chengdu 610041, China; qibiwuqie@163.com (W.Q.); 17882083378@163.com (J.Z.); wangylwy@163.com (Y.W.); liyanyan@swun.edu.cn (Y.L.); xiongyan0910@126.com (Y.X.); wangyong010101@swun.cn (Y.W.); 22100098@swun.edu.cn (W.L.); 2 Key Laboratory of Qinghai-Tibetan Plateau Animal Genetic Resource Reservation and Exploitation of Sichuan Province, Southwest Minzu University, Chengdu 610041, China; 3 College of Animal & Veterinary Science, Southwest Minzu University, Chengdu 610041, China; 4 College of Animal Science and Technology, Tarim University, Alar 843301, China; guishanxu@126.com

**Keywords:** goat, miR-92a-3p, intramuscular precursor adipocytes, adipocyte differentiation, *APOL*6

## Abstract

The quality of lamb meat is positively correlated with intramuscular fat content. In recent years, a large number of studies have shown that miRNAs play an important role in the proliferation and differentiation of adipocytes. In this study, we aimed to explore the effect of miR-92a-3p on the differentiation of goat intramuscular preadipocytes. The results showed that the expression level of miR-92a-3p was low in the early stage of differentiation, reached the highest level on the third day of differentiation, and then decreased. And miR-92a-3p can inhibit the accumulation of lipid droplets and down-regulate the determinants of adipogenic differentiation. Mechanistically, by predicting target genes, we found that miR-92a-3p affects the differentiation of goat intramuscular preadipocytes and the accumulation of lipid droplets by regulating the expression of goat gene *APOL6*. This study provides important new information to better understand the relationship between miRNAs and the differentiation of goat intramuscular preadipocytes, thus providing a new reference for goat intramuscular adipogenesis.

## 1. Introduction

Lamb meat is rich in essential amino acids, vitamins, and minerals, which makes it popular among consumers [1]. The content of intramuscular fat (IMF) is one of the key determinants of meat quality [2,3], and IMF has a great influence on the flavor of meat [4,5,6,7,8]. In recent years, there has been an increasing demand for lamb with high IMF content [9]. Intramuscular fat deposition in animals is a process in which preadipocytes accumulate fat and differentiate into mature adipocytes [10]. Adipocyte differentiation can be divided into two stages. In the differentiation decision stage, multifunctional stem cells differentiate directionally into adipose progenitor cells and further form precursor adipocytes. Subsequently, in the terminal differentiation stage, precursor adipocytes gradually differentiate into mature adipocytes with lipid droplets accumulation [11]. The differentiation process of precursor adipocytes is regulated by a complex network of transcription factors that orchestrate the expression of nearly one hundred proteins that are responsible for establishing the mature adipocyte phenotype. At the center of this network are two major adipogenic factors, peroxisome proliferator-activated receptor *γ* (*PPARγ*) and CCAAT/enhancer binding protein (*C/EBPα*), which oversee the entire process of terminal differentiation [12]. *PPARγ,* in particular, is recognized as a major regulator of adipogenesis [13]. In addition, preadipocyte differentiation is regulated by many signaling pathways [14]. For instance, adipocyte lipid metabolism-related genes *SREBP*1, *FAS,* and *LPL* have been shown to play a regulatory role in intramuscular fat deposition in goats.

MicroRNAs (miRNAs) are important post-transcriptional regulators of gene expression and play important roles in cell proliferation and differentiation, apoptosis, oncogene expression and inhibition, and lipid metabolism [15]. Many miRNAs have been found to regulate adipocyte differentiation and lipid deposition. For instance, in a study of 3T3-L1 cells, Li et al. found that miR-103 promoted adipogenesis in 3T3-L1 cells by targeting *MEF2D* [16]. In addition, another study reported that miR-429 promotes porcine preadipocyte differentiation and inhibits cell cycle progression by binding to the 3′UTR of *KLF9* and *p27* [17]. For ruminants, it has been shown that miR-27a regulates the synthesis and accumulation of lipid droplets in preadipocytes by targeting *CPT1B*, and that overexpression of *CPT1B* leads to a significant increase in lipid accumulation in sheep precursor adipocytes [18]. Moreover, in studies of subcutaneous fat, Shan et al. [19] found that miR-218-5p has an inhibitory effect in porcine preadipocyte differentiation by suppressing *ACSL1* expression. However, how miRNAs regulate intramuscular adipocyte differentiation in goats remains unclear.

*APOL*6 is a member of the apolipoprotein L (APOL) family and is a widely expressed lipid-binding protein [20]. Intracellular *APOL6* may affect the movement of lipids or allow lipids to bind to organelles [21]. *APOL6* is a lipid-binding protein with the structural domain of *BH3*, and lipid second messengers play an important role in initiating the apoptotic pathway; the current study suggests that *APOL6* plays an important role in apoptosis. However, no study has been reported on the role of *APOL6* in the differentiation of intramuscular adipocytes in goats. Our previous work showed that miR-92a-3p was differentially expressed before and after intramuscular adipocyte differentiation. Through the Target Gene Online Prediction Software, we found that there might be a targeting relationship between differentially expressed miR-92a-3p and *APOL*6. Whether miR-92a-3p affects intramuscular adipocyte differentiation by regulating *APOL*6 expression is unclear

In order to identify the miRNAs’ function in goat adipocyte, differentially expressed miR-92a-3p screened by whole transcriptome sequencing (RNA-seq) of goat intramuscular adipocytes during differentiation, was selected as the research object. In this study, we used overexpression, interferences, Bodipy and Oil red O staining, dual luciferase report assay, and qPCR to explore the effect of miR-92a-3p on the differentiation of goat intramuscular adipocytes. And we revealed that the inhibition of goat intramuscular preadipocyte differentiation by miR-92a-3p was mediated by targeting and regulating the expression of *APOL*6.

## 2. Materials and Methods

### 2.1. Cell Isolation, Culture, and Induction of Differentiation

This experiment complies with the requirements of ethical handling of experimental animals in China and meets the requirements of the Catalog of Ethical Handling of Experimental Animals in China. The samples were obtained from the intramuscular fat of the longissimus dorsi muscle of three 7-day-old male Jianzhou big-eared goats purchased from Sichuan Tiandi Sheep Industry Co., Ltd. (Chengdu, Sichuan, China). Goat intramuscular preadipocytes were isolated as described by Xu Q et al. [22]. DMEM-F12 (Gibco, Carlsbad, CA, USA) containing 10% fetal bovine serum, 1% penicillin-streptomycin (Gibco, Carlsbad, CA, USA), and 50 μmol·L^−1^ oleic acid (Sigma, St Louis, MO, USA) was used to induce intramuscular preadipocytes to differentiate into mature intramuscular adipocytes.

### 2.2. Quantitative Real-Time Polymerase Chain Reaction (qRT-PCR)

Total RNA was extracted according to the manufacture of Trizol (TaKaRa, Japan). The RNAs were then reverse transcribed using the RevertAid First Strand cDNA Synthesis Kit (Thermo, America) according to the protocol. Then, qPCR was performed using amplification primers with SYBR Green PCR Master Mix (TaKaRa). UXT or U6 were used as the internal control genes. The primer information is listed in Table 1.

### 2.3. Vector Construction

The mature sequence of goat miR-92a-3p was found on miRBase online program and compared with the laboratory sequencing results; after the results were consistent, the mimics and inhibitor of miR-92a-3p were synthesized according to the obtained mature sequence by Shanghai Gimo Pharmaceutical Technology Co. Named as m miR-92a-3p (miR-92a-3p mimics), i miR-92a-3p (miR-92a-3p inhibitor), and Negative Control were named NC (mimics NC) and iNC (inhibitor NC), respectively. The primers’ information is in Table 2.

The 3′UTR of *APOL6* was cloned by 30RACE kit (Takara, Tokyo, Japan): obtain the 3′UTR sequence of *APOL6* and analyze its cleavage site using DNAMAN software, and then combine with the sequence of pmirGLO vector. Two fast cleavage enzymes, *SacI* and *XhoI*, were selected for the double cleavage reaction. Double digestion system: 1 μg of gel recovery product (or 1 μg of pmirGLO plasmid), 1 μL of Q.cut *SacI*, 1 μL of Q.cut *XhoI*, 2 μL of 10 × Q.cut Buffer, supplemented with ddH_2_O up to 20 μL, and then purified after digestion at 37 °C for 0.5 h. After purified, the target fragment was ligated with T4 ligase with pmirGLO in a molar ratio of 5:1, and ligated for 10 h at 16 °C. After purification, the target fragment and pmirGLO were ligated with T4 ligase at a molar ratio of 5:1, and the constructed plasmid was named pmirGLO-*APOL*6 WT. According to the instructions of Mut Express ‖Fast Mutagenesis Kit V2 kit, the amplification primers of mutant vectors for miR-92a-3p were designed and named 92a-S and 92a-A (the information of the primers is shown in Table 3). The wild-type pmirGLO-*APOL*6 WT plasmid was used as the plasmid. The *APOL*6 WT plasmid was used as a template and the mutant vector plasmid, named pmirGLO-*APOL*6 92aMT, was obtained according to the kit instructions.

For construction of *APOL*6 overexpression vector, the primers were designed according to the sequence of *APOL*6 CDS region provided by NCBI (MK886491.1), and the primer sequences are shown in Table 3. The pM19T-*APOL*6 plasmid kept in the laboratory was used as the template, and amplified with the primers, the gel was recovered and purified, and the concentration was detected, then the plasmid was double cleaved by *NheI* and *NotI*, and at the same time the pcDNA-3.1 plasmid was double cleaved by the same enzyme. At the same time, the pcDNA-3.1 plasmid was double digested with the same enzyme, and the plasmid was extracted and stored at −20 °C; after the double digestion, it was verified that the vector was constructed correctly. Then, si-*APOL*6 was synthesized by Invitrogen. The primer information is listed in Table 3.

### 2.4. Luciferase Reporter Assaying

Resuscitate 293T cells, inoculate the F2 generation cells in 24-well plates, and perform co-transfection when the cell fusion reaches 60~70%. Four experimental groups were set up, and the specific groupings are shown in Table 4. Single-well co-transfection mix: 500 ng of vector plasmid, 2 μL of NC/miR-92a-3P mimics, and 2 μL of TurboFect transfection reagent added to 200 μL of Opti MEM for dilution and incubation. After 15 min of incubation, the reagent mixture was slowly added to each well of the cell culture plate, and after 12–20 h of transfection, the liquid mixture in the wells was discarded and replaced with complete medium. Then, 48 h later, the complete medium was aspirated off, rinsed gently twice with PBS, and 100 μL of 1 × PLB was added to each well, which was placed on the shaker at 180 r/min^−1^ for 30 min to fully lyse the cells, then the cells were lysed in accordance with the Dual-Luciferase^®^ Reporter Assay System with kit instructions followed to detect the reporter gene activity.

### 2.5. Lipid Droplets Staining

The main function of Bodipy staining and Oil Red O staining is to stain lipid droplets in the cells. And after Oil Red O staining, the OD value of the lipid droplets was measured by microplate reader to obtain the content of the lipid droplets, which is the quantitative result of Oil Red O staining.

Discard the medium in the 24-well plate with intramuscular adipocytes, rinse gently with PBS 2 times, fix with 10% formaldehyde solution for 30 min, discard the formaldehyde solution, rinse with PBS 2–3 times (try to rinse close to the wall), add 200 μL of Oil Red O working solution to each well (whichever is sufficient to cover the cells completely), stain for 30 min, then discard, and rinse with PBS several times until no precipitate can be seen in the wells, and then rinse with PBS. After no precipitate was visible, the 24-well plate was placed under a microscope to observe the morphology and number of lipid droplets in the cells. Finally, 1 mL of isopropanol was added to each well to extract intracellular lipids, and 200 μL of lipid extract was added to each well of a 96-well plate to measure the absorbance at 490 nm.

The Bodipy stock solution was prepared with distilled water at a ratio of 1:1000. The Bodipy stock solution was added to ddH_2_O and then quickly blown up with a pipette gun, and then immediately added into 24-well cell culture plates at 200 μL per well (avoiding light during the preparation). The cell culture plate was wrapped with tinfoil to protect it from light and placed under a microscope for rapid observation of the morphology and number of lipid droplets in goat intramuscular adipocytes.

### 2.6. Statistical Analysis

All data were presented as “means ± SD”. Variance of data was analyzed by SPSS 17.0. To assess the significance of differences between 2 groups, we used Student’s *t*-tests. Differences between multiple groups were assessed by one-way ANOVA analysis. * means the 0.01 ≤ *p* < 0.05, whereas ** means *p* < 0.01. All experiments in our study were carried out three times at least.

## 3. Results

### 3.1. Effect of Overexpression of miR-92a-3p on the Differentiation of Intramuscular Adipocytes in Goats

**Figure 1:**
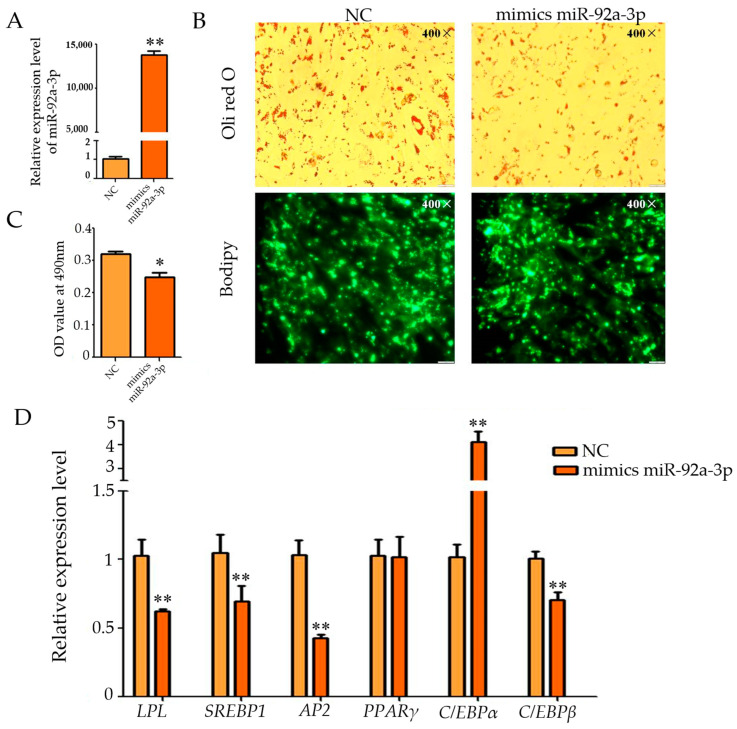
Effect of overexpression of miR-92a-3p on the differentiation of intramuscular adipocytes in goats (after transfection was completed, cells were collected after 48 h of oleic acid-induced differentiation and subjected to relevant tests and assays, * means the 0.01 ≤ *p* < 0.05, whereas ** means *p* < 0.01). (**A**): The overexpression efficiency of miR-92a-3p; (**B**): Oil red O staining and Bodipy staining figure (400×); (**C**): The result of OD value (490 nm); (**D**): The relative expression level of differentiation marker genes of goat intramuscular adipocytes after overexpressed miR-92a-3p.

The qPCR results showed that the expression of miR-92a-3p declined from 0 to 24 h during the differentiation of goat intramuscular adipocyte, whereas the expression gradually increased from 24 to 72 h, reaching a peak at 72 h of differentiation, and then the expression of miR-92a-3p gradually declined from 72 to 120 h of differentiation (Figure S1). Subsequently, miR-92a-3p mimics were utilized to up-regulate its expression in cells, and its overexpression efficiency was detected using qPCR technology. The results showed that mimics caused miR-92a-3p to be highly expressed in the m miR-92a-3p group (overexpression of miR-92a-3p group) and it was higher than that in the control group (*p* < 0.01, Figure 1A). Oil Red O and Bodipy staining showed that intracellular lipid droplets were significantly reduced during overexpression of miR-92a-3p (Figure 1B), and the real-time fluorescence quantitative PCR. The results were consistent with the above observations, and the OD value of the m miR-92a-3p group was significantly lower than that of the NC group (*p* < 0.05, Figure 1C). Furthermore, the relative expression of the marker genes of lipid differentiation, such as *LPL*, *AP*2, *SREBP*1, and *C/EBPβ,* were significantly inhibited during the overexpression of miR-92a-3p (all *p* < 0.01), while the expression of *C/EBPα* was highly promoted (*p* < 0.01), and no significant effect was found for the expression of *PPARγ* (*p* > 0.05, Figure 1D). These results suggest that overexpression of miR-92a-3p inhibits differentiation of goat intramuscular adipocytes.

### 3.2. Effect of miR-92a-3p Inhibition on Intramuscular Adipocyte Differentiation in Goats

**Figure 2:**
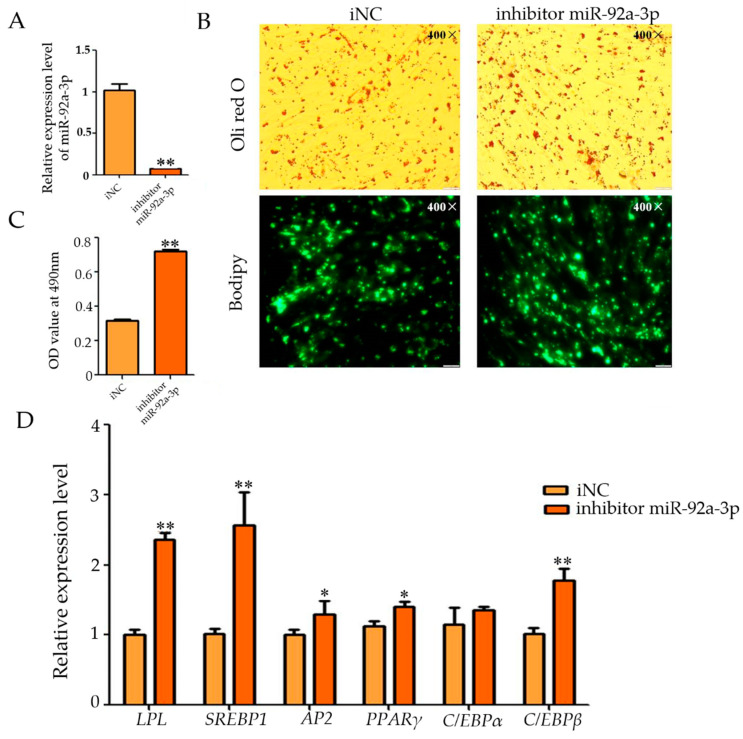
Effect of miR-92a-3p inhibition on intramuscular adipocyte differentiation in goats (after transfection was completed, cells were collected after 48 h of oleic acid-induced differentiation and subjected to relevant tests and assays, * means the 0.01 ≤ *p* < 0.05, whereas ** means *p* < 0.01). (**A**): The inhibiting efficiency of miR-92a-3p; (**B**): Oil red O staining and Bodipy staining figure (400×); (**C**): The result of OD value (490 nm); (**D**): The relative expression level of differentiation marker genes of goat intramuscular adipocytes after inhibited miR-92a-3p.

We synthesized the inhibitor based on the sequence of miR-92a-3p to inhibit its expression. The qRT-PCR results showed that the inhibitor successfully inhibited the expression of miR-92a-3p in the cells, and the miR-92a-3p expression in the experimental group was much lower than that in the control group (*p* < 0.01, Figure 2A). Oil Red O staining showed that inhibition of miR-92a-3p expression resulted in an increase in intracellular lipid droplets (Figure 2B), and qPCR results showed that the OD value of the i miR-92a-3p group (miR-92a-3p inhibition group) was highly significantly increased when compared with that of the iNC group (control group) (*p* < 0.01, Figure 2C). The qRT-PCR results showed that the expression levels of *LPL*, *SREBP*1, *C/EBPβ*, *AP*2, and *PPARγ* were significantly up-regulated after inhibiting the expression of miR-92a-3p (*p* < 0.05), whereas the relative expression levels of *C/EBPα* did not change significantly (Figure 2D). These results suggest that inhibition of miR-92a-3p promotes differentiation of goat intramuscular adipocytes.

### 3.3. Effect of miR-92a-3p on the Expression of APOL6

In order to further reveal the regulation mechanism of miR-92a-3p, we used TargetScan software to predict that APOL6 may be a target gene of miRNAs. And then, we further investigated the gene *APOL*6, and the relative expression levels of *APOL*6 were examined after miR-92a-3p expression was up-regulated or inhibited. The results showed that the expression level of *APOL6* was significantly decreased during overexpressing of miR-92a-3p (*p* < 0.01, Figure S2A), and the expression level of *APOL6* was significantly increased after miR-92a-3p inhibition (*p* < 0.01). To determine the targeting relationship between miR-92a-3p and *APOL*6, we performed a comparative analysis of the temporal expression profiles of miR-92a-3p and *APOL*6. The qPCR results showed that during the differentiation of goat intramuscular adipocytes, the expression level of *APOL*6 increased and then decreased from 0 to 120 h, increased at 96 h, and decreased at 96 h. The expression level of *APOL*6 in goat intramuscular adipocytes was also found to decrease from 0 to 120 h, and then increased at 96 h (Figure S2B).

### 3.4. miR-92a-3p Targets APOL6 3′UTR Binding

**Figure 3:**
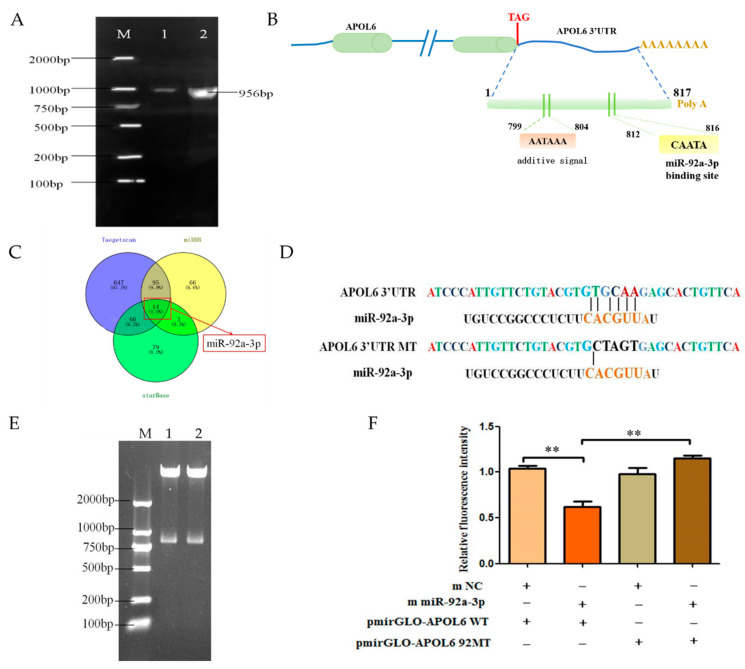
miR-92a-3p targets *APOL6* 3′UTR binding (** means *p* < 0.01). (**A**): PCR amplification result of *APOL*6 3′UTR in goat (M: DL2000 Marker; 1~2: *APOL*6 3′UTR); (**B**): *APOL*6 3′UTR sequence analysis diagram; (**C**): miRNAs might target *APOL*6 3′UTR; (**D**): *APOL*6 3′UTR wild-type and mutant sequence information; (**E**): Dual-luciferase identification results, M: DL2000 Marker, 1: pmirGLO-*APOL*6 WT, 2: pmirGLO-*APOL*6 92aMT; (**F**): Dual-luciferase reporter assay experiment results.

The sequence of goat *APOL*6 3′UTR was obtained by cloning using a 3′RACE kit, and the sequence length was 956 bp (Figure 3A), including 817 bp of *APOL*6 3′UTR, a poly A tail of 9 bp in length. Bioinformatics analysis of the obtained goat APOL6 3′UTR sequence revealed the presence of a classical plus-tail signal (AATAAA) at 799~804 bp of the goat *APOL*6 3′UTR, and the binding site of miR-92a-3p was located at 812~816 bp (Figure 3B). Using three different online programs (TargetScan, starBase, miRDB) to reverse predict the miRNAs that might target the goat *APOL*6 3′UTR, and using Veeny 2.1 to draw a Wayne diagram, the results showed (Figure 3C) that miR-92a-3p was located in the intersection of all three sets of software results. And the binding site of *APOL*6 3′UTR with miR-92a-3p was predicted using the above software (Figure 3C). In this study, we first constructed a wild-type dual luciferase reporter vector for *APOL*6 3′UTR (pmirGLO-*APOL*6 WT) and mutated miR-92a-3p at the predicted binding site of miR-92a-3p and *APOL*6 3′UTR (Figure 3D), and successfully constructed a mutant vector (pmirGLO-*APOL*6 92aMT). The two constructed vectors were identified by gel electrophoresis after double digestion, and the results showed that the size of the inserted fragment was the same as the length of the *APOL*6 3′UTR (Figure 3E), and the sequencing results also showed that the mutation of the site was successful, which proved that the two vector vectors were constructed successfully.

The results of the dual luciferase reporter gene detection system showed (Figure 3F) that transfection of miR-92a-3p mimics in goat intramuscular adipocytes, along with the pmirGLO-*APOL*6 WT plasmid, resulted in a highly significant reduction in the activity of the 3′UTR of *APOL*6 (*p* < 0.01); co-transfection of miR-92a-3p in goat intramuscular adipocytes with miR-92a-3p mimics and pmirGLO-*APOL*6 92aMT plasmid, on the other hand, had no significant effect on *APOL*6 3′UTR activity.

### 3.5. Effect of Overexpression of APOL6 on the Differentiation of Intramuscular Adipocytes in Goats

**Figure 4:**
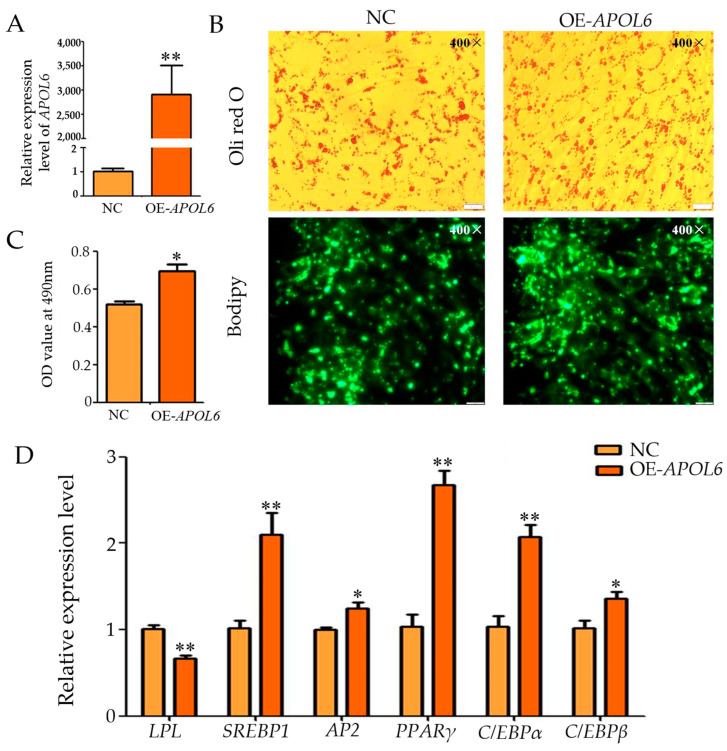
Effect of overexpression of *APOL*6 on the differentiation of intramuscular adipocytes in goats (after transfection was completed, cells were collected after 48 h of oleic acid-induced differentiation and subjected to relevant tests and assays, * means the 0.01 ≤ *p* < 0.05, whereas ** means *p* < 0.01). (**A**): *APOL*6 overexpression efficiency; (**B**): Oil red O staining and Bodipy staining figure (400×); (**C**): The result of OD value (490 nm); (**D**): The relative expression levels of goat intramuscular adipocyte differentiation marker genes after overexpression of *APOL6*.

The qPCR results showed that after transfection with the overexpression vector plasmid, the expression level of *APOL*6 in the experimental group’s cells was significantly higher than the control group’s cells (*p* < 0.01, Figure 4A), and Oil red O and Bodipy staining showed a significant increase in lipid droplets (Figure 4B). The OD value of the OE-APOL6 group was significantly increased compared to that of the Negative Control group (control group) (*p* < 0.05, Figure 4C). Meanwhile, qPCR results showed that the expression of *PPARγ*, *SREBP*1, and *C/EBPAα* was highly significantly up-regulated after overexpression of *APOL*6 (*p* < 0.01), and that the expression levels of *AP*2 and *C/EBPβ* were significantly up-regulated (*p* < 0.05), while the expression levels of *LPL* were significantly down-regulated (*p* < 0.01, Figure 4D). These results suggest that overexpression of *APOL*6 promotes differentiation of goat intramuscular adipocytes.

### 3.6. Effect of Inhibition of APOL6 on the Differentiation of Intramuscular Adipocytes in Goats

**Figure 5:**
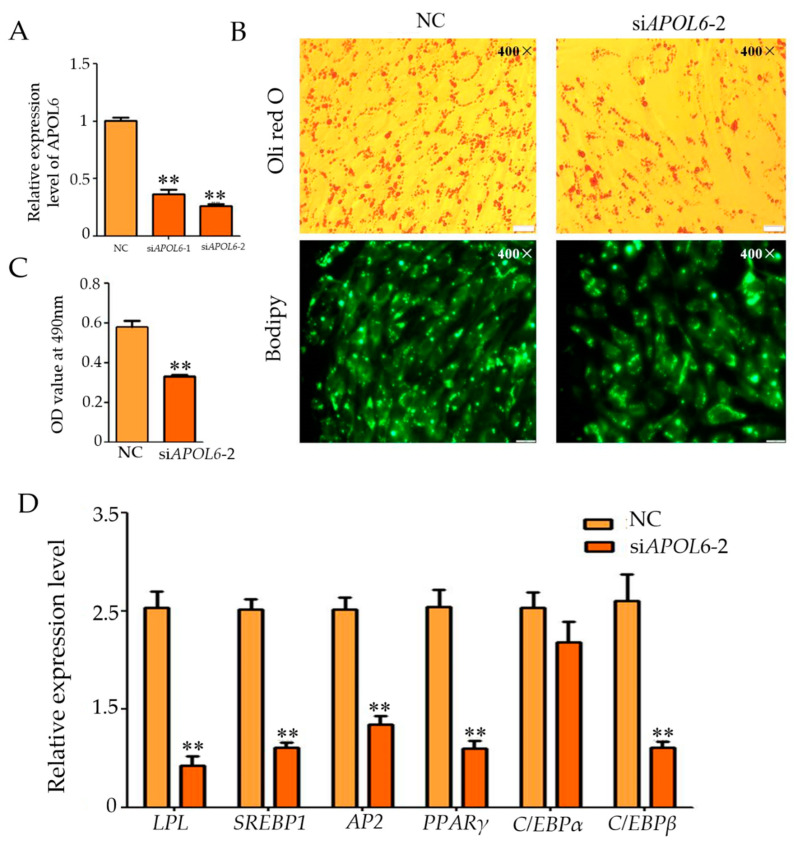
Effect of inhibition of *APOL*6 on the differentiation of intramuscular adipocytes in goats (after transfection was completed, cells were collected after 48 h of oleic acid-induced differentiation and subjected to relevant tests and assays, ** means *p* < 0.01). (**A**): The knockdown efficiency of *APOL*6; (**B**): Oil red O staining and Bodipy staining figure (400×); (**C**): The result of OD value (490 nm); (**D**): The relative expression levels of goat intramuscular adipocyte differentiation marker genes after knockdown of *APOL*6.

In order to further confirm the experimental result that overexpression of *APOL*6 promotes the differentiation of goat intramuscular adipocytes, interference experiments were also designed in this study. The qPCR results showed that the knockdown effect of specific siRNAs was good, and the expression of *APOL*6 was reduced by 64% in the si*APOL*6-1 group, and 74% in the si*APOL*6-2 group, compared with that of the NC group (Figure 5A). The interference effect of si*APOL*6-2 group was relatively better, so si*APOL*6-2 was chosen for the subsequent experiments. Oil red O and Bodipy staining results showed that compared with the NC group, the intracellular lipid droplets in the si*APOL*6-2 group were significantly reduced (Figure 5B), and the OD value at 490 nm was extremely significantly reduced (*p* < 0.01) (Figure 5C). The qPCR results showed that after interfering with *APOL6*, the relative expression levels of *PPARγ*, *LPL*, *C/EBPβ*, *AP2*, and *SREBP1* also decreased (*p* < 0.01, Figure 5D). These results suggest that inhibition of *APOL6* suppressed differentiation of goat intramuscular adipocytes.

### 3.7. Effect of Overexpression of miR-92a-3p Followed by Overexpression of APOL6 on the Differentiation of Goat Intramuscular Adipocytes

**Figure 6:**
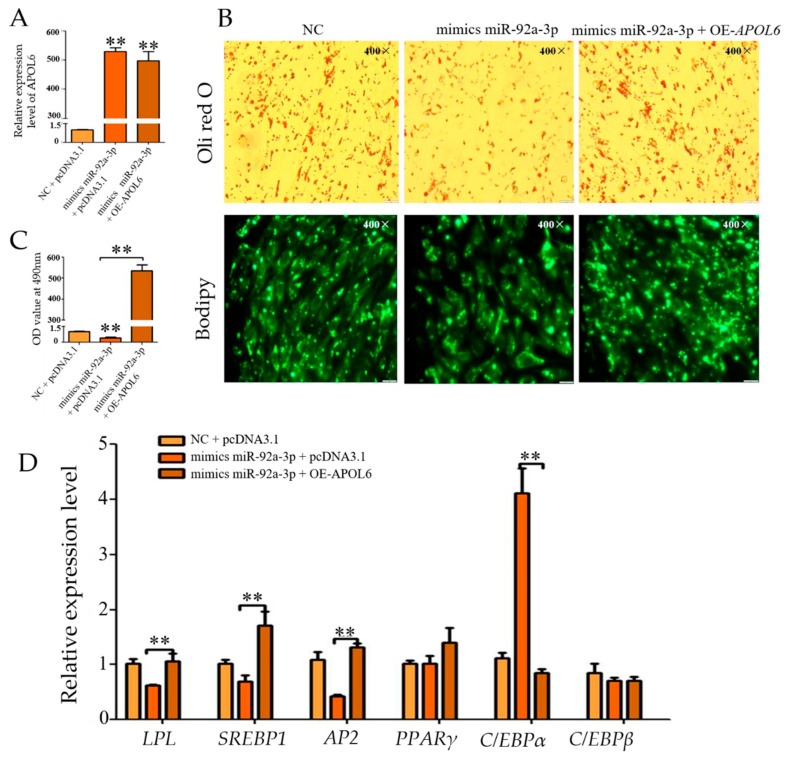
Effect of overexpression of miR-92a-3p followed by overexpression of *APOL*6 on the differentiation of goat intramuscular adipocytes (after transfection was completed, cells were collected after 48 h of oleic acid-induced differentiation and subjected to relevant tests and assays, whereas ** means *p* < 0.01). (**A**): The overexpression efficiency of miR-92a-3p; (**B**): Relative expression level of; (**C**): Oil red O staining and Bodipy staining figure (400×); (**D**): The relative expression levels of goat intramuscular adipocyte differentiation marker genes.

The qPCR results showed (Figure 6A) that the expression of miR-92a-3p was significantly increased in both the mmiR-92a-3p group and the mmiR-92a-3p + OEA group (*p* < 0.01). The qPCR results showed (Figure 6C) that compared with the m miR-92a-3p group, the expression of APOL6 in the m miR-92a-3p + OEA group increased significantly (*p* < 0.01). Oil Red O and Bodipy staining results showed (Figure 6B) that compared with the m miR-92a-3p group, the accumulation of intracellular lipid droplets in the m miR-92a-3p + OEA group increased. With overexpression of APOL6 after the overexpression of miR-92a-3p, the expression levels of SREBP1 and AP2 were significantly increased (*p* < 0.01), the expression of LPL was significantly increased (*p* < 0.05), and the expression of C/EBPβ was extremely significantly decreased (*p* < 0.01) (Figure 6D).

### 3.8. Effect of Inhibition of miR-92a-3p Followed by Interference with APOL6 on Goat Intramuscular Adipocyte Differentiation

**Figure 7:**
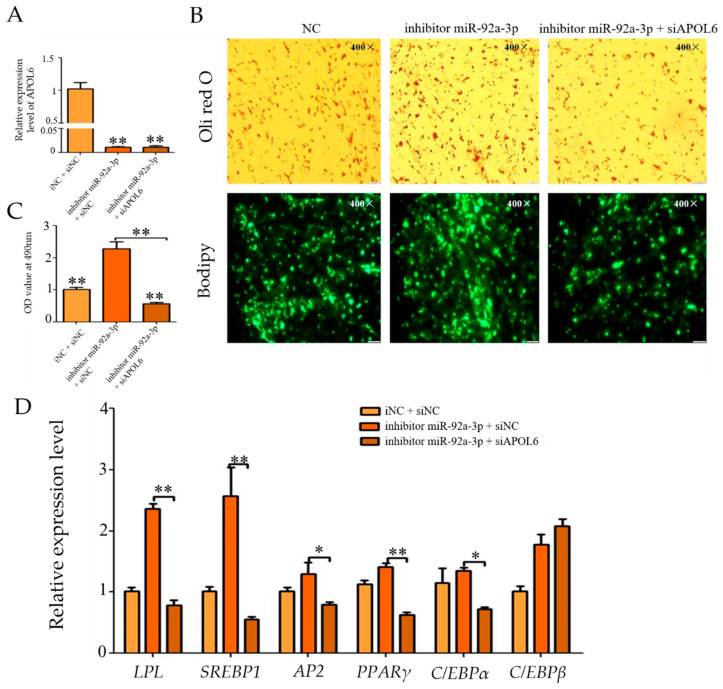
Effect of inhibition of miR-92a-3p followed by interference with APOL6 on goat intramuscular adipocyte differentiation (after transfection was completed, cells were collected after 48 h of oleic acid-induced differentiation and subjected to relevant tests and assays, * means the 0.01 ≤ *p* < 0.05, whereas ** means *p* < 0.01). (**A**): The inhibiting efficiency of miR-92a-3p; (**B**): Relative expression level of; (**C**): Oil red O staining and Bodipy staining figure (400×); (**D**): The relative expression levels of goat intramuscular adipocyte differentiation marker genes.

The qPCR results showed (Figure 7A) that the expression of miR-92a-3p was successfully suppressed in the i miR-92a-3p group versus the i miR-92a-3p + si*APOL*6 group (*p* < 0.01). Subsequently, the expression of *APOL*6 was detected, and the qPCR results showed (Figure 7B) that the expression of *APOL*6 in the i miR-92a-3p + si*APOL*6 group was extremely significantly decreased compared with that in the i miR-92a-3p group (*p* < 0.01). Oil red O and Bodipy staining results showed (Figure 7C) that intracellular lipid droplet aggregation was reduced in the i miR-92a-3p + si*APOL*6 group compared with the i miR-92a-3p group. After miR-92a-3p inhibition interfered with *APOL*6, the expression levels of *LPL*, *SREBP*1, and *PPARγ* were highly significantly down-regulated (*p* < 0.01), and the expression of *AP*2 and *C/EBPβ* was significantly decreased (*p* < 0.05, Figure 7D).

## 4. Discussion

Adipocyte differentiation is a complex biological process that involves numerous molecular events and requires the synergistic action of multiple factors such as transcription factors, functional genes, and signaling pathways. Ma et al. [23] found that miRNAs play a role in the transcriptional regulation of mammalian preadipocyte differentiation. The authors of [24] first demonstrated experimentally that miR-143 could promote adipocyte differentiation through mitogen-activated protein kinase 5, and subsequently, more and more miRNAs were experimentally verified to interact with transcription factors or signaling molecules important for adipocyte differentiation to regulate adipogenesis [25]. A large number of studies have found that miRNAs are involved in the differentiation process of adipocytes. From this, it is reasonable to speculate that miR-92a-3p may also be involved in the differentiation process of adipocytes in goat muscle. However, there is no report on the differentiation process of adipocytes in goat muscle. Therefore, in this experiment, we firstly constructed the cellular time-series expression profile of miR-92a-3p. Although the expression level of miR-92a-3p decreased in the late stage of cell differentiation, it was still higher than that in the pre-differentiation stage in general, so it was hypothesized that it might inhibit the differentiation of goat intramuscular adipocytes. However, its specific mechanism of action remains to be experimentally determined.

To further elucidate the exact role of miR-92a-3p in goat intramuscular adipocyte differentiation, we synthesized a mimic/inhibitor of miR-92a-3p and overexpressed/inhibited it in goat intramuscular adipocytes cultured in vitro, and the results showed that miR-92a-3p suppressed the production of lipid droplets, as well as down-regulating *LPL*, *AP*2, *SREBP*1, and *C/EBPβ* expression, while C/EBPα was significantly up-regulated. Among them, *C/EBPβ* is a positive regulator of adipocyte differentiation, and overexpression of *C/EBPβ* in 3T3-L1 precursor adipocytes or NIH-3T3 cells promotes adipogenic differentiation in the absence of hormone-induced differentiation [26]. Secondly, *PPARγ* plays a key role in adipocyte differentiation as a ligand-activated transcription factor [27]. *PPARγ* activation initiates adipocyte differentiation and lipogenic gene expression, leading to the synthesis and sustained accumulation of triglycerides, which in turn regulates a variety of biological processes in adipocytes from development to metabolism [28,29]. *SREBP*1, also known as adipocyte determinant and differentiation-dependent factor 1, is a transcription factor associated with adipocyte differentiation and compartmentalization. Studies have shown that *SREBP*1 plays a role in adipocyte gene expression by regulating the expression of *FAS* and *LPL*, important genes involved in fatty acid metabolism; meanwhile, *SREBP*1 also promotes adipogenic differentiation by inducing the expression of *PPARγ* [30]. Therefore, the significant down-regulation of *SREBP*1 and *C/EBPβ* may be the reason for the significant reduction in lipid droplets after overexpression of miR-92a-3p, and the up-regulation of the expression of *C/EBPα*, one of the factors promoting adipocyte differentiation, but after overexpression of miR-92a-3p, it may be due to the degree of inhibition of adipocyte differentiation after overexpression of miR-92a-3p is beyond the normal range, resulting in a kind of negative feedback regulation.

Apolipoprotein L (APOL) is a novel plasma protein belonging to the family of lipid-transporting proteins, and it is a novel high-density lipoprotein. Liu et al. [31] found that *APOL*6 overexpression could induce apoptosis in tumor cells and interact with lipid/fatty acid components in the apoptosis-inducing process, but *APOL*6 with the *BH*3 structure removed lost its apoptosis-inducing function. Zhaorigetu et al. [32] found that high expression of *APOL*6 induced apoptosis in cells such as macrophages, endothelial cells, and vascular smooth muscle cells, accelerating the progression of atherosclerosis. The role of *APOL*6 in adipocyte differentiation should not be ignored, Tan et al. [33] found that *APOL*6 is a target gene of miR-10b-5p, and the up-regulation of *APOL*6 expression after inhibition of miR-10b-5p also promoted the differentiation of 3T3-L1 cells, which suggests that *APOL*6 can play a role as a target gene of some miRNAs in the process of regulating adipocyte differentiation. However, no study has been reported on the role of *APOL*6 on the differentiation of intramuscular adipocytes in goats.

In this study, by constructing *APOL*6 overexpression vector and synthesizing siRNAs of *APOL*6, the results showed that overexpression of *APOL*6 promoted goat intramuscular adipocyte differentiation and intracellular lipid droplet production, while up-regulating the expression of *C/EBPβ*, *PPARγ*, *SREBP*1, and *AP*2, which was consistent with the finding of Tan et al. [34] that up-regulation of *APOL*6 expression promoted 3T3-L1 cell differentiation and adipogenesis; whereas, after interfering with *APOL*6, intracellular lipid droplet production was significantly reduced, while the expression of *LPL*, *C/EBPα*, *PPARγ*, *SREBP*1, and *AP*2 was significantly down-regulated, suggesting that down-regulation of *APOL*6 expression inhibited goat intramuscular adipocyte differentiation. Differentiated adipocytes were characterized as containing lipid droplets and expressing adipocyte marker genes such as *C/EBPβ*, *PPARγ*, *LPL*, lipocalin, and leptin [35]. It was found that *LPL* inhibited lipolysis, and the expression of *LPL* gradually increased during adipocyte differentiation, promoting the generation and accumulation of intracellular lipid droplets [36,37,38]; the inhibition of *APOL*6 resulted in a decrease in the expression of LPL and a reduction in intracellular lipid droplets, which was hypothesized to promote lipolysis through the inhibition of *LPL* expression, leading to a reduction in intracellular lipid droplets and the inhibition of goat intramuscular adipocyte differentiation. *C/EBPβ* is an early transcription factor that induces adipogenesis, and activates *PPARγ*, which is a major inducer of adipogenesis and a positive regulator of adipocyte differentiation, by regulating the transcription of its neighboring promoter [10]. *C/EBPα* has been shown to be a key gene in adipocyte differentiation in a previous study, and furthermore, it can regulate *AP*2 expression, which promotes the aggregation of lipid droplets, and can also combine with *C/EBPβ* and *PPARγ* to regulate the expression of fatty acid synthase (*FAS*). Taken together, *APOL*6 may promote adipocyte differentiation by promoting lipogenesis and by up-regulating the expression levels of *C/EBPβ*, *PPARγ*, *SREBP*1, and *AP*2, whereas interfering with *APOL*6 inhibits adipocyte differentiation and lipid droplet generation by repressing the expression of *LPL*, *C/EBPα*, *PPARγ*, *SREBP*1, and *AP*2. In this study, we found that overexpression of *APOL*6 promoted the differentiation of goat intramuscular adipocytes, suggesting that *APOL*6 positively regulates IMF deposition in goats. However, although the regulatory role of *APOL*6 in goat intramuscular adipocyte differentiation is unknown, the specific regulatory mechanisms need to be further investigated.

## 5. Conclusions

In summary, our study reveals that miR-92a-3p is a novel regulator of intramuscular preadipocyte differentiation in goats, and miR-92a-3p inhibits intramuscular preadipocyte differentiation in goats by targeting the *APOL6* gene. These findings contribute to a better understanding of miRNA-regulated adipogenesis.

## Figures and Tables

**Table 1:** Primer sequence information.

Gene/miRNA	Accession Number	Sequence (5′~3′)	Tm/°C	Purpose	Product Length
*U6*	S: TGGAACGCTTCACGAATTTGCG	NR_138,085.1	60	qPCR	68
	A: GGAACGATACAGAGAAGATTAGC				
*UXT*	S: GCAAGTGGATTTGGGCTGTAAC	XM_005700842.2	60	qPCR	180
	A: ATGGAGTCCTTGGTGAGGTTGT				
*miR-92a-3p*	S: CTGGAGTATTGCACTTGTCCCG		60	qPCR	
	A: GTGCAGGGTCCGAGGT				
*LPL*	S: TCCTGGAGTGACGGAATCTGT	NM_001285607.1	60	qPCR	156
	A: GACAGCCAGTCCACCACGAT				
*C/EBPα*	S: CCGTGGACAAGAACAGCAAC	XM_018062278	58	qPCR	142
	A: AGGCGGTCATTGTCACTGGT				
*PPARγ*	S: AAGCGTCAGGGTTCCACTATG	NM_001285658	60	qPCR	197
	A: GAACCTGATGGCGTTATGAGAC				
*SREBP1*	S: AAGTGGTGGGCCTCTCTGA	NM_001285755	58	qPCR	127
	A: GCAGGGGTTTCTCGGACT				
*C/EBPβ*	S: CAAGAAGACGGTGGACAAGC	XM_018058020.1	65	qPCR	204
	A: AACAAGTTCCGCAGGGTG				
*AP2*	S: TGAAGTCACTCCAGATGACAGG	NM_001285623.1	58	qPCR	143
	A: TGACACATTCCAGCACCAGC				

**Table 2:** Sequence information of mimics and inhibitor.

miRNA	Sequence	Classification
NC	UUCUCCGAACGUGUCACGUTT	mimics
	CAGUACUUUUGUGUAGUACAA	inhibitor
miR-92a-3p	UAUUGCACUUGUCCCGGCCUGU	mimics
	ACAGGCCGGGACAAGUGCAAUA	inhibitor

**Table 3:** The sequence information of overexpression and interference primers.

Gene	Primer Sequence (5′~3′)	Purpose
*APOL6*	S: CGGCTAGCATGGACAAAATGAGCATG	RT-PCR
	A: TTGCGGCCGCCTAAAATAGGAGCTGG	
*si-NC*	S: UUCUCCGAACGUGUCACGUTT	Interference
	A: ACGUGACACGUUCGGAGAATT	
*siAPOL6-1*	S: CAUGCCCUUGCAGACCACAUUGACA	Interference
	A: UGUCAAUGUGGUCUGCAAGGGCAUG	
*siAPOL6-2*	S: UGGCCGCUGGAAAGGUGAUUCAGAA	Interference
	A: UUCUGAAUCACCUUUCCAGCGGCCA	

**Table 4:** Experiment group.

Group Name	Treatment
NC WT	pmirGLO-APOL6 WT + mimics NC
NC MT	pmirGLO-APOL6 28MT + mimics NC
92a WT	pmirGLO-APOL6 WT + miR-92a-3p mimics
92a MT	pmirGLO-APOL6 92aMT + miR-92a-3p mimics

## Data Availability

The data used to support this study are available from the corresponding author on reasonable request.

## Supplementary Materials

The following supporting information can be downloaded at: https://www.mdpi.com/article/10.3390/genes15010057/s1, Figure S1: The temporal expression levels of miR-92a-3p in intramuscular adipocytes of goats; Figure S2. Effect of miR-92a-3p on the expression of APOL6 and Cellular temporal expression profile of APOL6. A: Effects of miR-92A-3p on APOL6 expression; B: The temporal expression levels of APOL6 in intramuscular adipocytes of goats.
