# Supplementary material for: Goat miR-92a-3p Targets APOL6 Gene to Regulate the Differentiation of Intramuscular Precursor Adipocytes
Source: Genes (Basel). 2023 Dec 30;15(1):57. doi: 10.3390/genes15010057 (PMC10815674; doi:10.3390/genes15010057)
Supplement: Supplementary file 1 [file genes-15-00057-s001.zip › genes-2791057-supplementary.pdf]

1. Cellular Temporal Expression Profile of miR-92a-3p

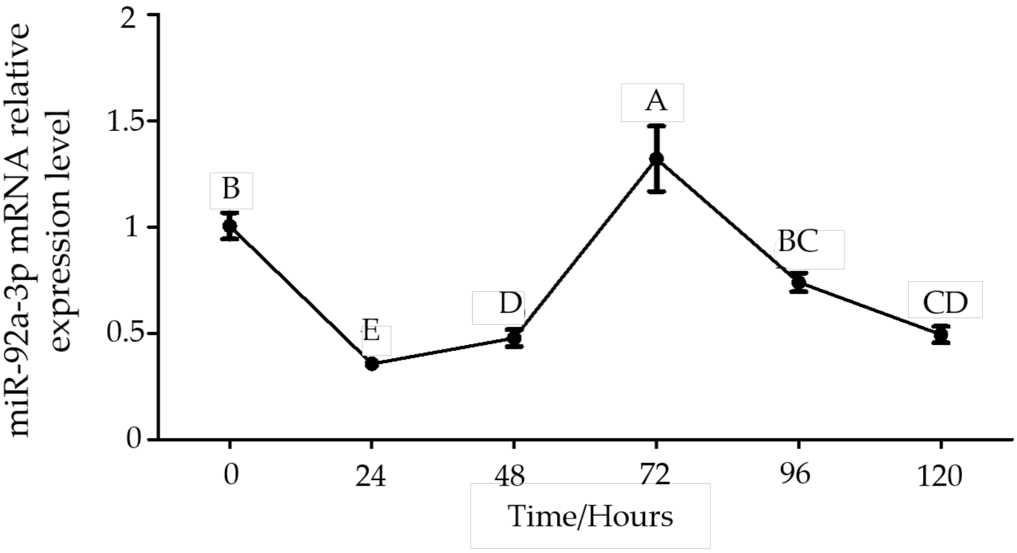

Figure S1. The temporal expression levels of miR-92a-3p in intramuscular adipocytes of goats.

2. Effect of miR-92a-3p on the Expression of *APOL6* and Cellular Temporal Expression Profile of *APOL6*

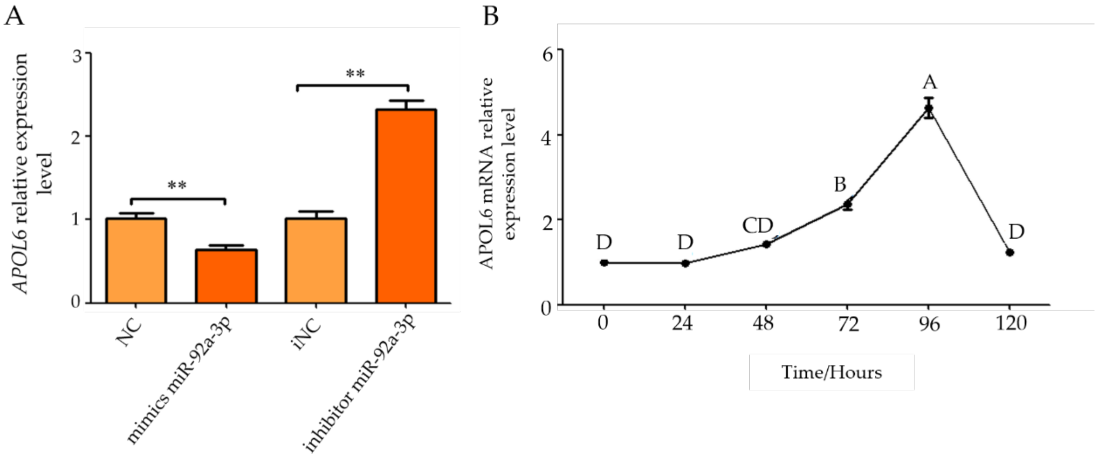

Figure S2. Effect of miR-92a-3p on the expression of *APOL6* and Cellular temporal expression profile of *APOL6*. A： Effects of miR-92A-3p on *APOL6* expression；B：The temporal expression levels of *APOL6* in intramuscular adipocytes of goats.
